# Molecular Mapping of QTLs Associated with Lodging Resistance in Dry Direct-Seeded Rice (*Oryza sativa* L.)

**DOI:** 10.3389/fpls.2017.01431

**Published:** 2017-08-21

**Authors:** Shailesh Yadav, Uma M. Singh, Shilpa M. Naik, Challa Venkateshwarlu, Perumalla J. Ramayya, K. Anitha Raman, Nitika Sandhu, Arvind Kumar

**Affiliations:** ^1^International Rice Research Institute, South Asia Hub, International Crops Research Institute for the Semi-Arid Tropics (ICRISAT) Patancheru, India; ^2^International Rice Research Institute Metro Manila, Philippines

**Keywords:** direct seeded rice, grain yield, lodging resistance, quantitative trait loci, SNP marker

## Abstract

Dry direct-seeded rice (DSR) is an alternative crop establishment method with less water and labor requirement through mechanization. It provides better opportunities for a second crop during the cropping season and therefore, a feasible alternative system to transplanted lowland rice. However, lodging is one of the major constraints in attaining high yield in DSR. Identification of QTLs for lodging resistance and their subsequent use in improving varieties under DSR will be an efficient breeding strategy to address the problem. In order to map the QTLs associated with lodging resistance, a set of 253 BC_3_F_4_ lines derived from a backcross between Swarna and Moroberekan were evaluated in two consecutive years. A total of 12 QTLs associated with lodging resistance traits [culm length (*qCL*), culm diameter (*qCD*), and culm strength (*qCS*)] were mapped on chromosomes 1, 2, 6, and 7 using 193 polymorphic SNP markers. Two major and consistent effect QTLs, namely *qCD*_1.1_ (with *R*^2^ of 10%) and *qCS*_1.1_ (with *R*^2^ of 14%) on chromosome 1 with id1003559 being the peak SNP marker (flanking markers; id1001973-id1006772) were identified as a common genomic region associated with important lodging resistance traits. *In silico* analysis revealed the presence of Gibberellic Acid 3 beta-hydroxylase along with 34 other putative candidate genes in the marker interval region of id1001973-id1006772. The positive alleles for culm length, culm diameter, and culm strength were contributed by the upland adaptive parent Moroberekan. Our results identified significant positive correlation between lodging related traits (culm length diameter and strength) and grain yield under DSR, indicating the role of lodging resistant traits in grain yield improvement under DSR. Deployment of the identified alleles influencing the culm strength and culm diameter in marker assisted introgression program may facilitate the lodging resistance under DSR.

## Introduction

Lodging in cereal crops is the result of the combined effects of the plants morphological traits and adverse weather conditions such as heavy winds and rains. Reduced crop yields, quality deterioration, and minimized harvesting efficiency are significantly associated with lodging (Kono, 1995). In recent years, due to limited water supply and escalating labor costs, direct-seeded rice (DSR) has become a major alternative among farmers in tropical countries (Sinniah et al., 2012). However, the large-scale cultivation of DSR is also prone to lodging, along with other problems such as uncontrolled weeds, yield reductions, and poor nutrient uptake (Nguyen and Ferrero, 2006). Moreover, lodging has been found to be more severe in DSR than in transplanted rice (Setter et al., 1997). Three types of lodging exist in cereal crops, namely: root lodging, stem bending, and stem breakage (Kono, 1995). Dry DSR is most susceptible to root lodging due to its shallow rooting system (Kobayashi et al., 2006; Hirano et al., 2014). Lowland rice is generally affected by the bending type of lodging due to the bending pressure of the upper internodes during strong winds and rains. Stem breakage occurs in the lower internodes of culms having a thin diameter and weak tensile strength (Islam et al., 2007).

Lodging resistance is a complex trait and influenced by many interacting agro-morphological traits. Different techniques have been used to measure the lodging effects in various crops (Kono, 1995; Watanabe, 1997), the most common of which is pushing the resistance of the lower part of the plant (Terashima et al., 1992; Won et al., 1998; Berry et al., 2003).

A number of investigations reported the culm diameter and thickness as the major factors contributing to lodging resistance (Ookawa and Ishihara, 1992; Zuber et al., 1999; Zhu et al., 2008; Ookawa et al., 2010). Various reports have found the correlation of culm strength with culm length, culm diameter, and yield in many cereal crops such as rice (Kashiwagi et al., 2008; Zhu et al., 2008), wheat (Keller et al., 1999; Zuber et al., 1999; Kelbert et al., 2004; Verma et al., 2005), maize (Flint-Garcia et al., 2003) and barley (Sameri et al., 2009).

The short stature of plants has earlier been the main target in improving the lodging resistance and harvest index of rice (Keller et al., 1999; Peng and Khush, 2003). However, statements cannot be generalized as the susceptibility of rice plant to lodging varies among cultivars with short plant height (Terashima et al., 1992). Reducing the plant's height also reduces its photosynthetic capacity and leads to a decrease in total biomass production, thus, restricting the plant's potential for further yield increase (Flintham et al., 1997; Murai et al., 2002; Okuno et al., 2014). The dwarfing gene (*sd*_*1*_) associated with short plant height has been used to show the negative pleiotropic effects on culm morphology in rice (Yano et al., 2015). Therefore, moderate plant height, large stem diameter, thick stem walls, and high lignin deposition have been recommended as the corrected preferential traits for the improvement of lodging resistance under DSR (Mackill et al., 1996).

Considering the current yield constraints under DSR conditions due to lodging, the identification of quantitative trait loci (QTLs) and candidate genes will increase our understanding of the regulatory mechanism of lodging resistance and will help breeders to improve lodging resistance under this system. A number of QTL mapping studies for culm length, strength, and thickness related to lodging resistance have been carried out using different rice segregating populations (Kashiwagi and Ishimaru, 2004; Mu et al., 2004; Kashiwagi et al., 2008; Zhu et al., 2008; Ookawa et al., 2010; Yano et al., 2015). By keeping all these points in mind, the present study aimed to (1) identify the genomic regions associated with lodging resistance, (2) examine the correlations of lodging resistance traits with grain yield, and (3) look into the possible candidate genes within the identified genetic regions.

## Materials and methods

### Plant materials and field experiments

The field experiments were conducted during the wet seasons (WS) of 2014 and 2015 at the International Rice Research Institute – South Asia Hub (IRRI-SAH), ICRISAT, Hyderabad, India (78° 16′longitude, 17° 32′ latitude with 540 m above the sea level). The experimental material consisted of a set of 253 lines derived from a backcross mapping population of Swarna^*^3/Moroberekan (Dixit et al., 2014). Genomic regions associated with early vigor and direct seeded rice related traits have been reported in same background in our previous study (Singh et al., 2017). The field trials were laid out in alpha-lattice design in two replications in the year 2014 and in augmented RCBD in the year 2015. Seeds were direct seeded in leveled, unpuddled soil under aerobic conditions by dibbling at a depth of 3 cm. Single row plot of 4 m were planted with row spacing of 20 cm. The pre-emergence herbicide Pendimethalin @1.5 ml/l was applied within 2 days after seeding (DAS) at surface moisture level. Manual weeding was followed regularly with 2 sprays of the post-emergence spray bispyribac-sodium (Nominee Gold) at 2 ml/l. The appropriate dosages of fertilizers and nutrients were administered during the critical stages of growth as recommended under the DSR system (Dingkuhn et al., 1990; Pal et al., 2008). The trials were irrigated once in a week throughout the cropping period. The tensiometers were installed across the field at 30 cm soil depth to measure soil moisture levels.

### Measurement of culm strength and other traits

At fully ripened stage, a prostrate tester (Daiki Rika Kogyou Co., Tokyo, Japan) was set perpendicular to the middle of the plant from 20 to 25 cm above the ground and the plant was pushed at an angle of 45° to measure the pushing resistance (Kashiwagi and Ishimaru, 2004). Culm strength (CS) was then estimated using the following formula (Hai et al., 2005):

$$\begin{array}{l} \text{Culm strength }\left(\text{gram}/\text{stem}\right)=\;\frac{\left(\frac{\text{test reading}}{40}\right)\;\times \;1000}{\text{No}.\text{ of tillers}} \end{array}$$

The culm strength was averaged over three plants for each line of the mapping population. Culm diameter (CD) in mm was measured using a sliding vernier caliper in the field from the same three plants at a height of 30 cm above ground level. Culm length (CL) in cm was measured as plant height minus panicle length (Mu et al., 2004). The other morphological traits viz., days to 50% flowering, plant height (cm), tiller number, panicle length (cm), and grain yield (kg ha^−1^) were recorded in both the years of 2014 and 2015. Days to 50% flowering (DTF) was recorded as when 50% of the panicles across the plot have emerged. At maturity plant height (cm) of three randomly chosen plants per plot was measured from ground level to the tip of the highest panicle and then averaged for analysis. The grain were harvested from each plot at physiological maturity, dried to moisture content ~14%, and then weighed to calculate the grain yield in kg ha^−1^. Tiller numbers were counted manually and panicle length was measured using a centimeter scale.

### Statistical analysis

Genotype means for each trial were estimated in the first stage analysis using the REML option of Proc Mixed (Littell et al., 2006), taking lines as fixed and replicates and blocks as random. In the second stage, a mixed model is fitted to the genotype-environment table of means and the weights estimated in the first stage (Frensham et al., 1997; Möhring and Piepho, 2009). The weights are the reciprocal of the variance of genotype means. The experimental error of each genotype-year combination is weighted by the variance of mean of the particular year. The mixed model is

$$\begin{array}{l} \gamma_{ij}=\;\mu_{j}+\;g_{i\;}+\;\omega_{ij} \end{array}$$

Where γ_*ij*_ is the adjusted mean of the *i*th genotype in the *j*th environment, μ_*j*_ is the main effect of the *j*th environment, *g*_*i*_ is the main effect of the *i*th genotype and ω_*ij*_ is the interaction of the *i*th genotype with the *j*th environment. Note that ω_*ij*_ subsumes residual error of the adjusted mean (Piepho, 2009). The residual variance is set to unity.

Year-wise correlations of lodging-associated traits with yield and other morphological traits such as days to 50% flowering, plant height (cm), tiller number, panicle length (cm), with grain yield (kg ha^−1^) were calculated using SAS PROC CORR (SAS Institute, 1992).

### Construction of linkage map and QTL identification for lodging resistance in rice

A total of 193 polymorphic SNPs were used to construct the linkage map by covering all 12 chromosomes spanning 1,525 cM with an average interval of 7.86 cM (Dixit et al., 2014) Phenotypic data on lodging associated traits were subject to QTL analysis to identify the genetic regions using QGene *ver* 4.3.10 (Joehanes and Nelson, 2008). The stepwise cofactor selection and default values were used for setting the CIM procedure in QGene. The genome scan interval was set to 1 cM and Window size was set at 10 cM. A LOD (logarithm of odds) score of 2.5 with 1,000 permutations was used for confirming the presence of a putative QTL. The QTLs responsible for phenotypic variance of more than 10% were considered as major-effect QTLs. The putative candidate genes for lodging-associated traits were identified based on the available literature and on the RAP database (http://rapdb.dna.affrc.go.jp/).

## Results and discussion

### Phenotypic evaluations of culm traits with lodging resistance

**Figure 1:**
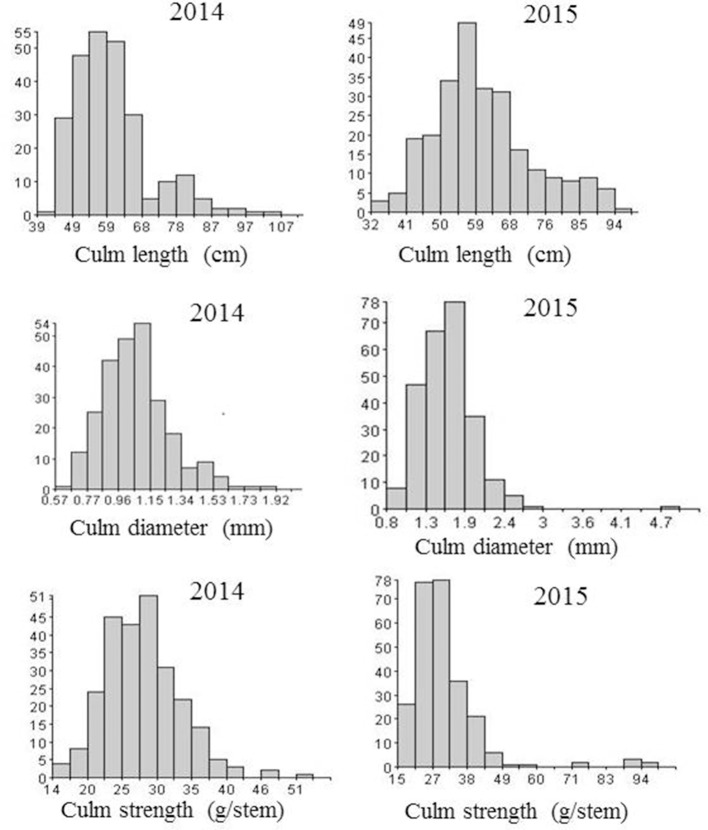
Frequency distribution of lodging-related traits in year 2014 and 2015.

Three culm traits associated with lodging resistance and other morphological traits such as plant height, days to 50% flowering, number of tillers, panicle length and grain yield were investigated. Analysis of variance revealed significant difference (*p* < 0.01) between the two parents (Swarna and Moroberekan) in all traits measured in both years. The overall progeny means for lodging-related traits were: culm length (59 ± 10.9 cm), culm diameter (1.65 ± 0.20 mm), and culm strength (28 ± 7.7 g/stem) with Moroberekan on the higher side in both years (Table 1). No transgressive segregant with a thicker diameter and higher culm strength than Moroberekan was observed. The phenotypic distribution of lodging-associated traits (CL, CD, and CS) was nearly normal in year 2014, while in 2015, CD and CS were skewed toward the Swarna (Figure 1). The culm strength of the progenies was classified from infirmness to good strength (good strength ≥ 40.1 gram/stem, medium 20.1~40 gram/stem, infirmness ≤ 20 gram/stem) on the basis of instrument reading (prostrate tester) and visual observation of plant lodging tendency correlated with the stem strength reading.

**Table 1:** Phenotypic means of the culm traits in 2 years.

**Trait**	**Year**	**Parent mean**		**Overall progeny mean**
		**Swarna**	**Moroberekan**	
Culm length (cm)	2014	49	91	60
	2015	47	86	59
Culm diameter (mm)	2014	0.9	1.8	1.04
	2015	1.3	4	1.65
Culm strength (g/stem)	2014	23.2	81.5	37
	2015	23.1	67.3	38

A two-stage analysis was used as the experimental design varied between the 2 years. The analysis indicated significant genotype × year interaction for grain yield, culm length and culm strength (Table 2). However the trial variance was 0 in the case of culm strength, culm length and grain yield and the genotype × year interaction was 0 in the case of culm diameter. The differences between genotypes were non-significant in the case of culm diameter. The best entries for grain yield, culm length and culm strength performing across the years are shown in Supplementary Table 1.

**Table 2:** REML parameters of the two-stage analysis across the two years (2014 and 2015).

**Variance components**		
	**Year**	**Gen × year**
Grain yield	0	1732276^**^
Culm strength	0	23.677^**^
Culm length	0.5577 (NS)	53.255^**^
Culm diameter	0.1798 (NS)	0
**Fixed effect**	**Gen**	
Grain yield	1.61^**^	
Culm strength	2.92^**^	
Culm length	3.60^**^	
Culm diameter	0.15 (NS)	

** *indicates significance at 1% level, NS indicates non-significance*.

### Phenotypic correlation between culm strength and other related traits

In both years, yield exhibited a moderate positive significant correlation with culm diameter and culm length however; it had a weak positive correlation with culm strength (Table 3). At the same time, culm strength and culm diameter were positively and significantly correlated (*r* = 0.682, *p* < 0.01 and *r* = 0.722, *p* < 0.01 in year 2014 and 2015, respectively). Earlier reports on various cereals including rice (Duan et al., 2004; Kashiwagi et al., 2008; Zhu et al., 2008), wheat (Li et al., 2000; Tripathi et al., 2003; Wang et al., 2006) and barley (Sameri et al., 2009) have reported significant associations of lodging resistance with culm diameter and the wall thickness of basal internodes. These findings indicate that increasing the culm diameter can improve the resistance of cereals to lodging and subsequently increase yields. It was also observed that the lines with yields greater than 5,000 kg ha^−1^ possessed intermediate culm length (59–70 cm) in 2014 and moderate to large culm length (51–90 cm) in 2015. The high yielding lines also possessed moderate measurements for culm length, strength, and diameter (Table 4).

**Table 3:** Correlation coefficients between 8 agro-morphological characters in 253 genotypes evaluated in 2 years (2014 and 2015).

	**DTF**	**PHT**	**NT**	**PL**	**CLEN**	**CDIAM**	**CSTR**	**GYKGPHA**
**2015 WS**								
DTF	1							
PHT	0.084(NS)	1						
NT	0.157^**^	−0.120^*^	1					
PL	−0.0471(NS)	0.68^**^	0.009(NS)	1				
CLEN	−0.097(NS)	0.981^**^	−0.152^**^	0.570^**^	1			
CDIAM	−0.151^**^	0.307^**^	−0.526^**^	0.255^**^	0.268^**^	1		
CSTR	−0.0676(NS)	0.181^**^	−0.329^**^	0.165^**^	0.165^**^	0.722^**^	1	
GYKGPHA	−0.023(NS)	0.382^**^	−0.047(NS)	0.383^**^	0.340^**^	0.218^**^	0.025 (NS)	1
**2014 WS**								
DTF	1							
PHT	−0.276^**^	1						
NT	0.112^*^	−0.225^**^	1					
PL	−0.222^**^	0.771^**^	−0.188^**^	1				
CLEN	0.142^**^	0.221^**^	−0.529^**^	0.17^**^	1			
CDIAM	−0.272^**^	0.994^**^	−0.219^**^	0.699^**^	0.218^**^	1		
CSTR	0.002(NS)	0.315^**^	−0.554^**^	0.258^**^	0.310^**^	0.682^**^	1	
GYKGPHA	−0.226^**^	0.361^**^	−0.103(NS)	0.401^**^	0.338^**^	0.122^*^	0.112^*^	1

* p < 0.05,

** *p < 0.01, NS, not significant; DTF, Days to 50% flowering; PHT, plant height; NT, number of tillers; PL, panicle length; CLEN, culm length; CDIAM, culm diameter; CSTR, culm strength; GYKGPHA, grain yield in kg ha^−1^*.

**Table 4:** Means of promising lines for culm length, culm diameter, and culm strength with grain yield in both the years 2014 and 2015.

**Promising lines**	**Grain yield (kg/ha)**		**Culm length (cm)**		**Culm diameter (mm)**		**Culm strength (g/stem)**	
	**WS 2014**	**WS 2015**	**WS 2014**	**WS 2015**	**WS 2014**	**WS 2015**	**WS 2014**	**WS 2015**
IR91648–B–29–B	5,717	5,208	80	89	0.9	2	30.2	31.3
IR91648–B–66–B	4,960	5,567	50	48	1	2.5	32.9	33.1
IR91648–B–104–B	5,611	5,450	58	52	1.3	1.7	38.7	33.8
IR91648–B–122–B	5,046	5,642	63	61	1.1	2.9	36.4	39.8
IR91648–B–124–B	5,182	7,183	65	63	1.2	1.7	31	29.9
IR91648–B–148–B	5,009	7,917	60	70	1.4	2	32.2	31.7
IR91648–B–200–B	5,160	5,425	60	63	1.2	2.3	35.3	40.3
IR91648–B–210–B	5,374	7,000	61	65	1.4	3	33.9	42.7
IR91648–B–238–B	5,368	7,900	64	60	0.9	2.2	33.8	30
IR91648–B–344–B	4,769	4,725	68	50	1.6	1.9	40.2	34.7
IR91648–B–367–B	4,350	5,075	62	68	1.5	2	35.3	37.5
Swarna	4,031	3,422	49	47	0.9	1.3	23.2	23.1
Moroberekan	3,138	2,788	91	86	1.8	4.0	81.5	67.3
Trial mean	3,684	3,675	60	59	1	1.66	27.7	27.51
LSD	1,768	2,419	8.51	15.12	0.42	2.52	11.2	47.24

### QTL detection

The introduction of the semi-dwarfing genes *rht*_*1*_ in wheat (Peng et al., 1999) and *sd*_*1*_ in rice (Sasaki et al., 2002) improved lodging resistance and increased yield significantly. However, to further improve yield, an increase of 10–20 cm in the height of the presently cultivated semi-dwarf rice varieties has been suggested as a viable alternative (Kumar et al., 1999). Therefore, identifying QTLs related to culm traits, together with increased plant height, is needed for improving lodging resistance and yield (Chen et al., 2014).

**Figure 2:**
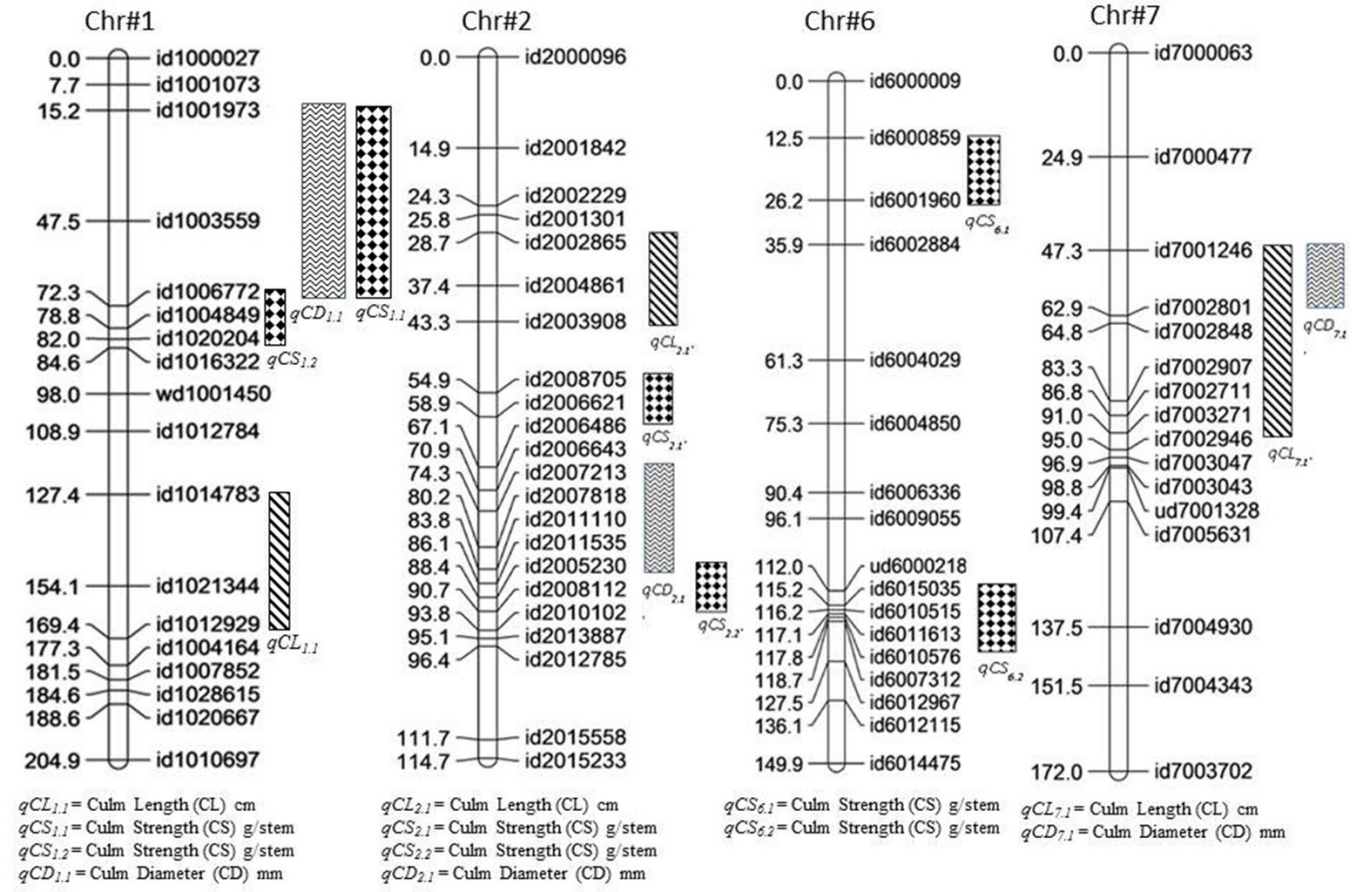
Positions of the QTLs identified for culm length (*qCL*), culm diameter (*qCD*) and culm strength (*qCS*) derived from backcross mapping population (Swarna^*^3/Moroberekan).

In the present study, 12 QTLs controlling culm length, culm diameter, and culm strength were detected over four different chromosomes in the backcross population of Swarna^*^3/Moroberekan (Figure 2, Table 5). Chromosome 1 harbored the maximum number of QTLs for lodging-associated traits. A total of three culm length QTLs (LOD>3.0) were detected on chromosomes 1, 2, and 7 (*qCL*_1.1_, *qCL*_2.1_, and *qCL*_7.1_) over the 2 years. The allele for higher culm length for *qCL*_2.1_ was from the taller parent Moroberekan while the semi-dwarf parent Swarna contributed favorable alleles to the other two loci (*qCL*_1.1_ and *qCL*_7.1_). The explained phenotypic variance ranges from 11 to 26.0%. Previously, Mu et al. (2004) identified six additive QTLs associated with culm length on chromosomes 2, 3, 4, 5, and 6 with a phenotypic variation of 39% collectively. Matsubara et al. (2016) mapped four QTLs for culm length on chromosome 1, 2, 5, and 6. In present study, the QTL *qCL*_*1.1*_ identified at physical position of 36.1 Mbp on chromosome 1 with 26% of phenotypic variance was 4 Mbp closer from large effect QTL for culm length (with *R*^2^ of 73%) reported by Matsubara et al. (2016).

**Table 5:** QTLs identified for culm length, culm diameter, and culm strength in backcross population.

**Trait**	**QTLs**	**Peak marker**	**Position (cM)**	**Marker interval**	**LOD value**		***R***^**2**^**%**		**Additive effects^*a*^**	
					**2014**	**2015**	**2014**	**2015**	**2014**	**2015**
Culm Length (CL) cm	*qCL_*1.1*_*	id1021344	154.10	id1014783–id1012929	17	–	26	–	–3.8	-
	*qCL_*2.1*_*	id2004861	37.40	id2002865–id2003908	7	–	12	–	9.8	-
	*qCL_*7.1*_*	id7002801	62.9	id7001246–id7002946	–	2.5	–	11	-	–1.5
Culm Diameter (CD)mm	qCD_1.1_^*^	id1003559	47.50	id1001973–id1006772	8.3	2.9	14	10	0.8	0.19
	*qCD_*2.1*_*	id2007818	80.20	id2007213–id2005230	–	8.3	–	13	-	0.65
	*qCD_*7.1*_*	id7001246	47.3	id7001246–id7002801	2.6	–	12	–	0.4	-
Culm Strength (CS) g/stem	qCS_1.1_^*^	id1003559	47.50	id1001973–id1006772	6.7	2.8	23	14	0.8	0.22
	*qCS_*2.1*_*	id2007818	80.20	id1006772–id1020204	3.5	–	12	–	1.4	-
	*qCS_*2.2*_*	id2006621	58.90	id2008705–id2006486	–	2.9	–	27	-	3.9
	*qCS_*2.3*_*	id2008112	90.7	id2005230–id2010102	–	3.2	–	31	-	4
	*qCS_*6.1*_*	id6001960	26.2	id600859–id6001960	2.8	–	–	11	3	-
	*qCS_*6.2*_*	id6010515	116.2	id6015035–id6010576	–	2.5	–	8	-	1.8

* *Indicates locus detected both in 2014 and 2015; “–” indicates locus was not detected in that particular year*.

a *Positive additive effect value indicates that the allele is contributed by MO (Moroberekan)*.

Three QTLs that affected culm diameter were detected on chromosomes 1 (*qCD*_1.1_), 2 (*qCD*_2.1_), and 7 (*qCD*_7.1_) with phenotypic variance explained by 10–14%. The alleles for culm diameter (*qCD*_1.1_, *qCD*_2.1_, and *qCD*_7.1_) were contributed by Moroberekan. The QTL *qCD*_1.1_ was consistent in both 2014 and 2015 with a phenotypic variance of 14 and 10%, respectively. Many researchers have detected QTLs for higher culm diameter on chromosome 1 at physical interval of 6Mbp-11 Mbp using various mapping populations (Kashiwagi and Ishimaru, 2004; Mu et al., 2004; Yano et al., 2015). The culm diameter was reported to have positive effects on the section modulus (SM) and bending moment at breaking (M), leading to lodging resistance without any yield loss (Ookawa et al., 2010). Keller et al. (1999) identified two QTLs for culm thickness associated with lodging resistance in a population between wheat and spelt. The previous reports and present findings demonstrate the role of culm diameter in enhancing lodging resistance in rice.

Six QTLs (*qCS*_*1.1*_, *qCS*_*2.1*_, *qCS*_*2.2*_, *qCS*_*2.3*_, *qCS*_*6.1*_, and *qCS*_*6.2*_) for culm strength were detected on three chromosomes in the present study. The QTLs with positive effect for culm strength were contributed by the Moroberekan parent. Previously, a number of QTLs *viz., prl5, SCM1, SCM2, SCM3*, and *SCM4* were reported for culm strength (Kashiwagi and Ishimaru, 2004; Ookawa et al., 2010; Yano et al., 2015). However, some of the identified QTLs region was quite large for its utilization in marker-assisted selection; fine mapping, sequencing and allele mining of the identified regions could be useful in further deployment. The QTL *qCS*_*1.1*_ identified in present study was reproducible in both 2014 and 2015 with a phenotypic variance of 23 and 14%, respectively and located on chromosome 1. Mu et al. (2004) also reported QTL for culm strength on same chromosome in marker interval of RM5-RM302. The QTLs *qCS*_*6.1*_ and *qCS*_*6.2*_ were identified on chromosome 6 with phenotypic variance of 11 and 8%, respectively. The QTL *qCS*_*6.2*_ for culm strength identified in the present study was in agreement with earlier reported QTL *STRONG CULM2* (*SCM2*) at physical position of 27 Mbp (Ookawa et al., 2010). The detailed analysis of *SCM2* through positional cloning and sequencing reveals that the gene is similar to *APO1* favorably leads to increase in the spikelet number and enhanced culm strength by increasing the cell proliferation rate (Ookawa et al., 2010). The QTL-hotspot for early vigor, early uniform emergence (*qEV*_*6.1*_, *qEUE*_*6.1*_) in interval of 11.7-27.6 cM under DSR conditions reported by Singh et al. (2017) was located near to QTL for culm strength (*qCS*_*6.1*_) identified in present study at position of 26.2 cM. The observed direct relationship among the seedling establishment and lodging resistant traits in term of co-location of genetic regions could be an important landmark for various desirable traits under DSR conditions.

### Co-localization of QTLs

In this study, the stable and consistent effect QTL region between id1001973-id1006772 markers reported to be associated with the traits culm diameter and culm strength indicating the pleiotropic effect of the identified genomic region or linkage of loci controlling both the traits. Current finding in the present study are in agreement with the previous report of lodging resistant traits in rice (Kashiwagi and Ishimaru, 2004; Mu et al., 2004). The identified shared loci can be fine mapped and cloned to resolve the underlying gene functions constituting the QTL region (Eshed and Zamir, 1995; Ashikari et al., 2005; Pelgas et al., 2011). Though the genetic distance of this co-localized region is of considerable length (47.5–72.3 cM), it may provide an important clue for future efforts to explore the region by fine mapping to better understand the mechanism of this complex trait.

### Identification of putative candidate genes

The genomic regions within the consistent QTL for culm diameter (*qCD*_1.1_) and culm strength (*qCS*_1.1_) were analyzed *in silico* for the presence of possible candidate genes previously reported for lodging resistance. A total of 35 putative genes were found within the stable QTL region identified for culm-associated traits (Table 6). Many genes within the QTL were responsible for phytohormones synthesis, hypothetical and expressed protein, heat shock protein, transcriptional factors, and precursors for various biochemical and metabolic pathways. Among them, a putative candidate gene Gibberellic Acid (GA) 3 beta-hydroxylase with single copy 4003659-4004946 bp was identified. This gene is the final precursor in the biosynthetic pathway of the plant hormone Gibberellin (Itoh et al., 1999). Another report (Okuno et al., 2014) highlighted the effect of the plant hormone GA on lodging resistance in rice and on increased total biomass signifying the positive impact of over-expression of GA on lodging resistance due to increased culm diameter and lignin deposition. GA was also found to increase biomass yield. A few other findings reported that higher lignin content is responsible for improved varietal resistance to the bending type of lodging (Ookawa and Ishihara, 1993; Biemelt et al., 2004). Recently, Ookawa et al. (2016) also discussed the effect of high GA level results in enlargement of culm diameter by increasing the number of parenchymatous cells in the culm. Further studies needed to confirm the positive regulation of GA on culm thickness in the genomic region on chromosome 1. Such candidate genes need to be further characterized to be used as potential targets for marker-assisted breeding for strong/thick culm providing lodging resistance in rice.

**Table 6:** List of putative genes found within the stable QTL on chromosome1 in the marker interval of id1001973–id1006772.

**Locus id**	**Position Start–End (bp)**	**Description**
Os01g0174200	3819067–3819782	Conserved hypothetical protein.
Os01g0174300	3820168–3824523	NADH: cytochrome b5 reductase (CBR) family protein.
Os01g0174500	3827039–3830326	Prolyl 4–hydroxylase, alpha subunit domain containing protein.
Os01g0174600	3830492–3831846	Zinc finger, CCCH–type domain containing protein.
Os01g0174700	3835129–3838211	Similar to Akt (Fragment).
Os01g0174900	3845541–3842753	Glutaredoxin–related protein family protein.
Os01g0175000	3846181–3849671	Phospholipase/Carboxyl esterase family protein.
Os01g0175100	3855046–3856060	Kv1.4 voltage–gated K+ channel family protein.
Os01g0175200	3858824–3861839	Tetratricopeptide–like helical domain containing protein.
Os01g0175300	3870734–3874321	Ankyrin repeat containing protein.
Os01g0175500	3878068–3875761	Protein of unknown function DUF1618 domain containing protein.
Os01g0175600	3878069–3900085	${\text{HCO}}_{3}^{--}$ transporter, eukaryote family protein.
Os01g0176200	3926556–3924363	UDP–glucuronosyl/UDP–glucosyltransferase family protein.
Os01g0176300	3927450–3929447	Protein prenyltransferase domain containing protein.
Os01g0177100	3984922–3976431	Similar to STYLOSA protein.
Os01g0177200	3995398–3986612	Similar to Ubiquitin–specific protease 14.
Os01g0177400	4003659–4004946	GA 3 beta–hydroxylase.
Os01g0177900	4033751–4037473	ABC–2 type transporter domain containing protein.
Os01g0178000	4051856–4057690	Aminotransferase, class I and II domain containing protein.
Os01g0178500	4073676–407643	Similar to Auxin–responsive protein (Aux/IAA) (Fragment).
Os01g0178600	4082680–4081316	Peptidase A1, pepsin family protein.
Os01g0178700	4094859–4091068	Similar to Membrane associated protein with a RING finger, 4xtransmembrane domain, transcripts identified by EST.
Os01g0179200	4122579–4118041	Syntaxin/epimorphin family protein.
Os01g0179300	4126379–4123420	Conserved hypothetical protein.
Os01g0179500	4150414–4143351	Conserved hypothetical protein.
Os01g0179600	4152864–4154680	UDP–glucuronosyl/UDP–glucosyltransferase family protein.
Os01g0179700	4160946–4165165	Similar to GTP–binding protein YPTM2.
Os01g0179700	4160946–4165165	Similar to GTP–binding protein YPTM2.
Os01g0179800	4167511–4171559	TMS membrane protein/tumor differentially expressed protein family protein.
Os01g0180000	4179159–4181434	Pistil–specific extensin–like protein family protein.
Os01g0180300	4208553–4213302	Lipoprotein, type 6 family protein.
Os01g0180400	4215032–4217547	Protein of unknown function DUF581 family protein.
Os01g0180600	4234514–4244531	Similar to MutS homolog 7 (Fragment).
Os01g0180700	4245417–4249851	Conserved hypothetical protein.
Os01g0180800	4259626–4253838	Heat shock protein Hsp70 family protein.

The QTL for culm strength (*qCS*_6.2_) identified in the present study was also explained by Ookawa et al., 2010 by identifying candidate gene *SCM2* within the marker interval of RM 20546–RM 20562 at physical location of 27 Mbp. This gene was found to be responsible for the physical strength of the culm and for the increase in spikelet number because of pleiotropic gene action. This finding points to the authenticity of the QTL hot spot region for lodging resistance genes and will be an important landmark in future breeding programs for improved lodging resistance in rice.

## Conclusion

Culm diameter and culm strength are potential target traits for breeding rice varieties with enhanced lodging resistance under DSR conditions. The stable and consistent genetic region within the marker interval of id1001973-id1006772 could be an important source of alleles responsible for enhancing lodging resistance in rice. The polymorphic SNP markers in these QTL regions may be utilized for future marker-assisted breeding programs. Functional validation of the candidate gene *SCM2* as well as genes for GA biosynthesis with precursors of various metabolic pathways associated with culm QTLs (*qCS*_*6.2*_, *qCS*_*1.1*_, and *qCD*_*1.1*_) will be a valuable future step to clearly understand the mechanism of lodging resistance.

## Author contributions

SY was involved in conducting the experiment, recording observations and drafting the article; US, SN, CV, and PR helped in experimental work and contributed to the manuscript modification; KR, NS was involved in experimental analysis, interpretation of data and revising the manuscript; AK was involved in the design of the experiment and in the critical revision of the manuscript. All authors approved the final version of the manuscript.

### Conflict of interest statement

The authors declare that the research was conducted in the absence of any commercial or financial relationships that could be construed as a potential conflict of interest.

## Abbreviations

DSR: Direct seeded rice
CL: Culm length
CD: Culm diameter
CS: Culm strength
SNP: Single nucleotide polymorphism
CIM: Composite Interval Mapping
PCA: Principal component analysis
SAS: Statistical Analysis System.
