# Supplementary material for: Molecular Mapping of QTLs Associated with Lodging Resistance in Dry Direct-Seeded Rice (Oryza sativa L.)
Source: Front Plant Sci. 2017 Aug 21;8:1431. doi: 10.3389/fpls.2017.01431 (PMC5567065; doi:10.3389/fpls.2017.01431)
Supplement: Supplementary file 1 [file Table1.DOC]

| **ENTRY** | **Grain yield (kg/ha)** | **Culm diameter (mm)** | **Culm length (cm)** | **Culm strength (g/stem)** |
| --- | --- | --- | --- | --- |
| IR91648-B-104-B | 5485.52 | 1.4436 | 54.79 | 36.22 |
| IR91648-B-122-B | 5350.62 | 2.4214 | 61.99 | 38.11 |
| IR91648-B-148-B | 6431.91 | 1.6872 | 65.27 | 31.93 |
| IR91648-B-174-B | 5018.78 | 1.5244 | 58.97 | 29.21 |
| IR91648-B-200-B | 5295.85 | 1.9537 | 61.51 | 37.81 |
| IR91648-B-210-B | 6199.21 | 2.5213 | 63.17 | 38.31 |
| IR91648-B-29-B | 5369.98 | 1.6329 | 84.19 | 30.71 |
| IR91648-B-31-B | 5281.07 | 1.5125 | 75.22 | 28.91 |
| IR91648-B-291-B | 5402.28 | 1.2794 | 72.35 | 21.74 |
| IR91648-B-59-B | 5918.76 | 1.1875 | 66.745 | 26.06 |
| IR91648-B-83-B | 5719.59 | 1.4639 | 58.41 | 23.68 |
| IR91648-B-92-B | 6139.68 | 1.0897 | 61.53 | 25.08 |
| IR91648-B-89-B | 6045.92 | 1.4296 | 62.87 | 27.71 |
| MOROBEREKAN | 2990.63 | 3.0555 | 88.91 | 74.39 |
| SWARNA | 3734.26 | 1.07 | 47.71 | 23.16 |
| Overall trial mean | 3661.92 | 1.36 | 59.69 | 27.62 |

**Table 1 Mean performances of genotypes based on the two-stage combined analysis**

**across WS2014 and WS2015**
